# Supplementary material for: MetaRibo-Seq measures translation in microbiomes
Source: Nat Commun. 2020 Jun 29;11:3268. doi: 10.1038/s41467-020-17081-z (PMC7324362; doi:10.1038/s41467-020-17081-z)
Supplement: Supplementary file 10 — Supplementary Data 7 [file 41467_2020_17081_MOESM10_ESM.zip › File2/Confidence_VeryHigh_Taxonomy/425858_out.krona.html]

Javascript must be enabled to view this page.

members
magnitude
magnitudeUnassigned
count
unassigned
taxon
rank

425858\_out

4

4
2
superkingdom

1
phylum
976

class
117743
1

200644
order
1

1
49546
family

1
1016
genus


SRS015596\_contig\_number\_23401
1019
species
1

1
phylum
1239

1
species
1263017

SRS023526\_contig\_number\_contig-100\_244.203171

2
phylum
544448

2
class
31969

2
186329
order

2

SRS012969\_contig\_number\_32130SRS077392\_contig\_number\_37933
species
2053494
